# The impact of muscle function, muscle mass and sarcopenia on independent ageing in very old Swedish men

**DOI:** 10.1186/s12877-019-1142-y

**Published:** 2019-05-29

**Authors:** Kristin Franzon, Björn Zethelius, Tommy Cederholm, Lena Kilander

**Affiliations:** 1 Department of Public Health and Caring Sciences/Geriatrics, Uppsala University, Box 564, 751 22 Uppsala, Sweden; 2 Department of Public Health and Caring Sciences/Clinical Nutrition and Metabolism, Uppsala University, Uppsala, Sweden

**Keywords:** Sarcopenia, EWGSOP1, EWGSOP2, Muscle mass, Muscle function, Gait speed, Hand grip strength, Chair stand test, Independent ageing

## Abstract

**Background:**

Preserved functions of daily life and cognition are cornerstones of independent aging, which is crucial for maintaining a high quality of life. The aim of this study was to examine the impact of sarcopenia, and its underlying components, on independent ageing in a cohort study of very old men.

**Methods:**

The presence of sarcopenia and independent ageing at a mean age of 87 was investigated in 287 men from the Uppsala Longitudinal Study of Adult Men. Five years later 127 men were re-evaluated for independent ageing. Sarcopenia was defined by two different definitions from the European Working Group on Sarcopenia in Older People. In the first definition sarcopenia was defined as skeletal muscle index < 7.26 kg/m^2^ and either gait speed ≤0.8 m/s or hand grip strength < 30 kg. In the later up-dated definition, HGS < 27 kg and/or chair stand test > 15 s defines probable sarcopenia, which is confirmed by SMI < 7.0 kg/m^2^. Independent ageing was defined as a Mini-Mental State Examination score of ≥25 points, absence of diagnosed dementia, community-dwelling, independency in personal care and ability to walk outdoors alone.

**Results:**

Sarcopenia at baseline was observed in 21% (60/287) and 20% (58/287), respectively, due to definition. The prevalence of independent ageing was 83% (239/288) at baseline and 69% (87/127) five years later. None of the sarcopenia diagnoses were associated with independent ageing. In contrast, gait speed was both in cross-sectional (odds ratio (OR) per one standard deviation increase 2.15, 95% confidence interval (CI) 1.47–3.15), and in longitudinal multivariate analyses (OR 1.84, 95% CI 1.19–2.82). In the cross-sectional analysis also higher hand grip strength was associated with independent ageing (OR 1.58, 95% CI 1.12–2.22), while a slower chair stand test was inversely associated (OR 0.61, 95% CI 0.43–0.86). Muscle mass; i.e. skeletal muscle index, was not associated with independent ageing.

**Conclusions:**

For very old men, especially a higher gait speed, but also a higher hand grip strength and a faster chair stand test, were associated with independent ageing, while skeletal muscle index alone, and the composite sarcopenia phenotype measured with two different definitions, were not.

## Background

Very old individuals value functional independence higher than absence of morbidity [1]. This health valuation is in line with our concept of independent ageing [2, 3], defined as preserved ability to perform personal activities of daily living (ADL), walking outdoors and cognitive function. Thus, independent ageing requires preserved function of the neuromuscular system. With this in mind, sarcopenia can be one reason for not achieving independent ageing. Sarcopenia was initially defined as low muscle mass only [4], but now there is an agreement that low muscle mass must be combined with low muscle function to define sarcopenia [5–8]. However, how to measure muscle mass or function is still an open question [5–8]. In 2010, the European Working Group on Sarcopenia in Older People (EWGSOP) defined sarcopenia as low appendicular skeletal muscle mass and low gait speed (GS) and/or hand grip strength (HGS) [5]. Recently this definition was up-dated, when low muscle strength was further emphasized as a key characteristic of sarcopenia [8]. In the EWGSOP2 definition, low muscle strength, measured by HGS and/or chair stand test (CST), is defined as probable sarcopenia. Sarcopenia is then confirmed by low appendicular skeletal muscle mass, while tests of physical performance (e.g. gait speed (GS)) add information of the severity of the sarcopenia. Studies using the updated sarcopenia definition are so far scarce [9, 10].

In two meta-analyses, sarcopenia was associated with functional decline and cognitive impairment, respectively [11, 12]. However, studies on individuals ≥80 years are rare. Two studies have investigated the association between sarcopenia and cognitive impairment [13, 14], but to our knowledge there has been no previous study on this age group concerning the association between sarcopenia and ADL function or outdoor walking. Furthermore, studies in this field with functional and cognitive impairment as a combined outcome are rare for all ages [15–18].

The aim of this study was to investigate the cross-sectional and longitudinal associations between sarcopenia defined by the two EWGSOP definitions and independent ageing, and among their respective components. A cohort of Swedish men with a mean age of 87 at baseline and with follow-up five years later was used.

## Methods

### Study population

**Fig. 1:**
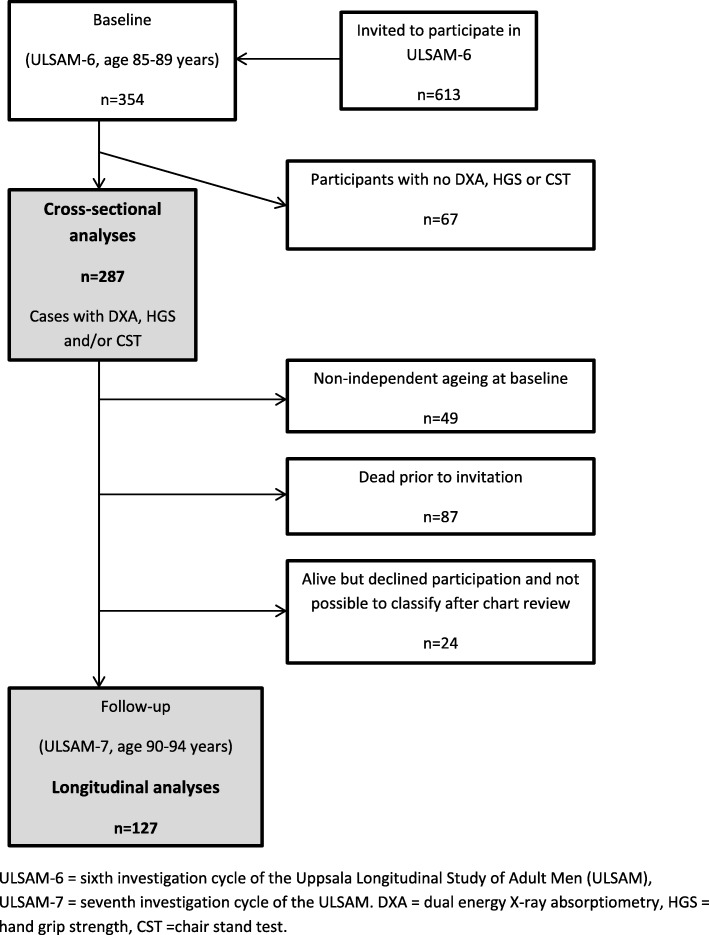
Flow chart of study participants. ULSAM-6 = sixth investigation cycle of the Uppsala Longitudinal Study of Adult Men (ULSAM), ULSAM-7 = seventh investigation cycle of the ULSAM. DXA = dual energy X-ray absorptiometry, HGS = hand grip strength, CST = chair stand test

The Uppsala Longitudinal Study of Adult Men (ULSAM) started in 1970 [19]. All men born in the period 1920–24 and living in Uppsala were invited to the study, and 82% (*n* = 2322) participated in the first investigation at the age of 50. The baseline for the present study was the investigation conducted in 2008–09 at a mean age of 87 (*n* = 354) (Fig. 1). Of these participants, it was possible to define 287 men with regard to both independent ageing and sarcopenia. No exclusion criteria were applied. Forty-nine participants did not fulfil the criteria for independent ageing at baseline and 87 men had died before follow-up. Thus, 105 participants were re-examined in 2013–15 at a mean age of 92. Another 46 men declined to participate but it was possible to re-evaluate 22 of these concerning dementia, living conditions and ADL after a review of their medical records.

The study was approved by the Regional Ethical Review Board at Uppsala University, and all subjects provided written informed consent.

### Sarcopenia and covariates at baseline

Body composition, including total fat mass, was measured by dual energy X-ray absorptiometry (DXA) using a DPX Prodigy, Lunar corp., Madison, WI, USA. Skeletal muscle index (SMI) was calculated by dividing the sum of the lean mass in the arms and legs by height squared (kg/m^2^). GS was assessed in 284 men using a 10-m course. Participants were instructed to walk at a comfortable speed, and GS was derived from the middle 6 m. If needed, an assistive device was allowed. HGS was measured in 285 men using a Baseline® hydraulic hand dynamometer. Both hands were measured three times and the highest value was used. CST was assessed in 244 men. The participant was asked to rise five times from a seated position with arms folded across the chest, and the time needed was measured. Sarcopenia was defined using both the old (2010) and the new (2018) definitions proposed by the EWGSOP [5, 8]. According to EWGSOP1, sarcopenia was defined as SMI < 7.26 kg/m^2^ and GS ≤0.8 m/s and/or HGS < 30 kg [5]. In the EWGSOP2 definition, probable sarcopenia was defined by low muscle strength, i.e. HGS < 27 kg and/or CST > 15 s [8]. Low muscle strength together with SMI < 7.0 kg/m^2^ confirmed the sarcopenia diagnosis and severe sarcopenia was present if GS ≤0.8 m/s.

Educational level was classified as low [< 8 years], medium [8–13 years], or high [> 13 years]). At baseline a valid questionnaire [20, 21] was used to obtain information on leisure-time physical activity: sedentary (mainly reading or watching television), moderate (walking outdoors or cycling regularly), regular (sports or strenuous gardening ≥3 h per week) and athletic (regular strenuous physical activity). Participants were categorised as living with someone (spouse/cohabitant, other relatives) or not. Participants who smoked at baseline and/or at the fifth investigation cycle at mean age 82 were categorised as smokers. The National Patient Registry provided information on in-patient care before baseline, and this information was used to calculate Charlson’s Comorbidity Index [22, 23]. Weight and height were measured by a research nurse, and body mass index (BMI) was calculated (kg/m^2^).

### Independent ageing

Independent ageing, at baseline and follow-up, was defined as Mini-Mental State Examination [24] (MMSE) ≥25 points, absence of diagnosed dementia, community-dwelling, independence in personal ADL and ability to walk outdoors alone. An experienced research nurse administered the MMSE to 287 men at baseline and 95 men at follow-up. Two geriatricians independently determined dementia status according to best practice and pre-specified criteria [25–29] using all data available in the medical records from Uppsala University Hospital, primary care and nursing homes in Uppsala County until April 1, 2009 and January 1, 2015, respectively. A questionnaire including questions on living conditions, ADLs (bathing, dressing, toileting) and ability to walk outdoors (assistive device allowed) were answered by 211 men at baseline and 104 men at follow-up. The medical records were reviewed for consistency with the self-reported information, and these provided supplemental information on participants not answering the questionnaire. Data on living conditions were provided by the Swedish Population Register. The medical records of non-participants at follow-up were also reviewed and it was possible to classify another 22 men, except regarding performance on the MMSE, and include them in the longitudinal analysis (Fig. 1).

### Statistical methods

Continuous variables are presented as means and standard deviations (SD) and categorical variables as the number of individuals and percentages. We used logistic regression to calculate the odds ratios (ORs) and 95% confidence intervals (CIs) of the association of the exposure variables with independent ageing and its components.

SMI, GS, HGS and CST are presented as continuous variables standardised so that the estimates indicate the OR per SD increase in the variable. The covariates included in the multivariable model were age, smoking status, Charlson comorbidity index and total fat mass. The statistical software package JMP 13 for PC (SAS Corporation, Cary, NC, USA) was used for all analyses.

## Results

The baseline characteristics are presented in Table 1. The prevalence of sarcopenia was 21% according to the original EWGSOP definition. With the updated EWGSOP2 definition, the prevalence of probable sarcopenia was 73%, confirmed sarcopenia 20% and severe sarcopenia 2%. Six percent displayed GS ≤0.8 m/s and 61% performed the chair stand test in > 15 s. Close to half (46%) had HGS below 30 kg and 28% below 27 kg. The prevalence of independent ageing was 83% at baseline, and 69% five years later (Table 2). Those who were re-examined (*n* = 127) had a higher prevalence of never smoking and less morbidity than the total population (*n* = 287). Table 2 presents the prevalence of the different components of independent ageing. At baseline, 99% of the participants were community-dwelling and 95% were free from dementia.

**Table 1:** Baseline characteristics of the populations used for the cross-sectional and longitudinal analyses, respectively

	Total population in cross-sectional analyses *n* = 287	Participants who were followed up in longitudinal analyses n = 127
Age (years), mean ± SD	86.6 ± 1.0	86.5 ± 1.0
Educational level, n (%)		
Low	140 (49)	56 (44)
Medium	88 (31)	44 (35)
High	59 (21)	27 (21)
Physical activity, n (%)		
Sedentary	49 (24)	13 (14)
Moderate	82 (40)	42 (47)
Regular	69 (33)	30 (33)
Athletic	6 (3)	5 (6)
Living with someone, n (%)	137 (65)	67 (73)
Smoking status, n (%)		
Smoker	16 (6)	4 (3)
Former smoking	155 (54)	64 (50)
Never smoking	116 (40)	59 (46)
Charlson Comorbidity Index, n (%)		
0	111 (39)	60 (47)
1	104 (36)	51 (40)
≥ 2	72 (25)	16 (13)
Body mass index, kg/m^2^, mean ± SD	25.6 ± 3.3	25.8 ± 3.0
Total fat mass, kg, mean ± SD	22.2 ± 7.5	22.8 ± 7.4
EWGSOP1		
Sarcopenia, n (%)	60 (21)	25 (20)
EWGSOP2		
Probable sarcopenia, n (%)	209 (73)	81 (64)
Confirmed sarcopenia, n (%)	58 (20)	24 (19)
Severe sarcopenia, n (%)	5 (2)	1 (0.8)
Skeletal muscle index, kg/m^2^, mean ± SD	7.5 ± 0.8	7.5 ± 0.7
< 7.0 kg/m^2^	72 (25)	34 (27)
< 7.26 kg/m^2^	109 (38)	49 (39)
Gait speed, m/s, mean ± SD	1.4 ± 0.3	1.5 ± 0.3
≤ 0.8 m/s	16 (6)	2 (2)
Hand grip strength, kg, mean ± SD	30.2 ± 6.5	31.2 ± 6.1
< 27 kg	79 (28)	32 (25)
< 30 kg	132 (46)	53 (42)
Chair stand test, s, mean ± SD	17.9 ± 7.1	16.4 ± 6.0
> 15 s	148 (61)	54 (48)

*SD* standard deviation, *EWGSOP* European Working Group on Sarcopenia in Older People

**Table 2:** Prevalence of Independent ageing and its indicators at baseline and 5 years later

	Total population at mean age 87 years	Participants who were followed up at mean age 92 years
	Cross-sectional analyses	Longitudinal analyses
	No of cases/no. of subjects (%)	
Independent aging	239/287 (83)	87/127 (69)
Mini-Mental State Examination score ≥ 25 p	252/287 (88)	85/95 (89)
No diagnosis of dementia	272/287 (95)	112/127 (88)
Community-dwelling	284/287 (99)	112/127 (88)
No assistance with personal care^a^	274/286 (96)	107/125 (86)
No assistance with outdoor walking	274/285 (96)	106/119 (89)

^a^Independent in bathing, dressing and toileting

Table 3 shows the adjusted cross-sectional associations between sarcopenia and independent ageing. Severe sarcopenia was associated with loss of independent ageing and needing assistance with outdoor walking. No other sarcopenia category was associated with independent ageing or its separate components. Higher GS and HGS were associated with independent ageing and no assistance with outdoor walking. Higher GS was also associated with MMSE ≥25 p and independency in personal care. Further, slower CST was inversely associated with independent ageing. There were no associations between SMI and independent ageing or the single components.

**Table 3:** Cross-sectional associations between sarcopenia and independent ageing at baseline, and between their respective components

	Independent aging	MMSE ≥ 25 p	No dementia	Community-dwelling	No assistance with personal care^b^	No assistance with outdoor walking
	*n* = 239	*n* = 252	*n* = 272	*n* = 284	*n* = 274	*n* = 274
	Odds ratio (95% Confidence interval)					
EWGSOP1						
Sarcopenia	1.08 (0.47–2.46)	1.63 (0.58–4.55)	n/a	n/a	2.16 (0.25–18.9)	0.37 (0.080–1.68)
EWGSOP2						
Probable sarcopenia	0.54 (0.23–1.24)	0.65 (0.26–1.60)	1.49 (0.41–5.35)	n/a	1.08 (0.20–5.79)	1.23 (0.22–6.88)
Confirmed sarcopenia	0.69 (0.31–1.50)	0.75 (0.31–1.82)	n/a	n/a	1.65 (0.19–14.6)	0.54 (0.099–2.98)
Severe sarcopenia	0.087 (0.013–0.59)	0.47 (0.048–4.50)	n/a	n/a	0.080 (0.0062–1.03)	0.015 (0.0013–0.17)
Skeletal muscle index^a^, kg/m^2^	1.05 (0.75–1.46)	0.97 (0.66–1.41)	1.11 (0.64–1.92)	0.48 (0.13–1.73)	0.87 (0.44–1.74)	0.97 (0.48–1.94)
Gait speed^a^, m/s	2.15 (1.47–3.15)	1.70 (1.14–2.56)	1.54 (0.82–2.89)	1.02 (0.23–4.52)	2.34 (1.10–5.02)	3.75 (1.59–8.85)
Hand grip strength^a^, kg	1.58 (1.12–2.22)	1.24 (0.80–1.80)	1.45 (0.83–2.56)	0.60 (0.17–2.14)	1.65 (0.85–3.18)	2.35 (1.16–4.77)
Chair stand test^a^, s	0.61 (0.43–0.86)	0.85 (0.57–1.26)	1.06 (0.49–2.29)	0.51 (0.031–8.22)	0.61 (0.37–1.01)	0.79 (0.41–1.51)

*EWGSOP* European Working Group on Sarcopenia in Older People, *MMSE* Mini-mental State Examination, *n/a* not applicable

Adjusted for age at baseline, smoking status, Charlson comorbidity index and total fat mass

^a^The results are showed as odds ratios per one standard deviation in continuous variables

^b^Assistance with bathing, dressing and/or toileting

Table 4 presents the adjusted associations between sarcopenia at baseline and independent ageing five years later. Higher GS was associated with maintained independent ageing and no need of assistance with personal care, while higher HGS was associated with still being community-dwelling. There were no associations between the different sarcopenia definitions, SMI or CST and subsequent independent ageing or its individual components.

**Table 4:** Longitudinal associations between sarcopenia at baseline and independent ageing five years later, and between their respective components

	Independent aging	MMSE ≥ 25 p	No dementia	Community-dwelling	No assistance with personal care^b^	No assistance with outdoor walking
	*n* = 87	*n* = 85	*n* = 112	n = 112	*n* = 107	*n* = 106
	Odds ratio (95% Confidence interval)					
EWGSOP1						
Sarcopenia	1.04 (0.40–2.74)	1.09 (0.20–5.86)	1.76 (0.35–8.78)	2.57 (0.45–14.7)	1.89 (0.39–9.07)	0.36 (0.10–1.31)
EWGSOP2						
Probable sarcopenia	0.56 (0.24–1.30)	0.80 (0.17–3.76)	0.26 (0.058–1.15)	0.39 (0.095–1.62)	0.61 (0.19–1.94)	0.55 (0.14–2.26)
Confirmed sarcopenia	1.14 (0.43–3.06)	2.19 (0.25–19.2)	1.79 (0.36–8.82)	1.74 (0.35–8.74)	1.22 (0.31–4.75)	1.18 (0.23–5.93)
Severe sarcopeni	n/a	n/a	n/a	n/a	n/a	n/a
Skeletal muscle index^a^, kg/m^2^	1.04 (0.71–1.53)	0.80 (0.41–1.56)	0.75 (0.42–1.34)	0.94 (0.53–1.66)	0.88 (0.52–1.48)	1.00 (0.55–1.83)
Gait speed^a^, m/s	1.84 (1.19–2.82)	1.15 (0.59–2.25)	1.63 (0.94–2.83)	1.35 (0.77–2.38)	2.34 (1.29–4.26)	1.21 (0.67–2.20)
Hand grip strength^a^, kg	1.43 (0.95–2.14)	0.91 (0.44–1.88)	1.39 (0.76–2.54)	1.94 (1.01–3.74)	1.37 (0.80–2.36)	1.44 (0.73–2.84)
Chair stand test^a^, s	1.01 (0.66–1.54)	1.35 (0.43–4.20)	1.09 (0.58–2.03)	0.88 (0.55–1.40)	1.04 (0.57–1.88)	1.10 (0.51–2.35)

*EWGSOP* European Working Group on Sarcopenia in Older People, *MMSE* Mini-mental State Examination, *n/a* not applicable

Adjusted for age at baseline, smoking status, Charlson comorbidity index and total fat mass

^a^The results are showed as odds ratios per one standard deviation in continuous variables

^b^Assistance with bathing, dressing and/or toileting

## Discussion

The major finding of this study was that higher GS and HGS and less time to perform CST were associated cross-sectionally with independent ageing in very old men. Higher GS at baseline was also associated with independent ageing five years later. Further, severe sarcopenia, only present in 2%, was associated with non-independent ageing in the cross-sectional analyses. However, there was no association between SMI or the other sarcopenia categories, as defined according to the EWGSOP1 and 2 criteria [5, 8], and independent ageing.

Regarding functional variables, both GS and HGS are well-established markers of biological ageing so the findings are not surprising [30–34]. However, studies of the oldest old with outcomes similar to ours are scarce [16, 18]. In the Honolulu Heart Program, high GS and HGS at mean age 76 was not associated with healthy ageing at age 85 [18]. Healthy ageing was defined as being free from major diseases, no physical limitation and with normal cognitive test performance [18]. However, in the cross-sectional Helsinki Businessmen Study, higher GS was correlated with active and healthy ageing in octogenarian men [16]. In contrast to the present study, the criteria for active and healthy ageing were self-reported and included “feeling happy”, absence of major diseases, functional or cognitive impairment [16]. Lower HGS was also correlated with the combination of physical and cognitive impairment in a cross-sectional study in participants with a mean age of 68 years [15]. To the best of our knowledge, there are, so far, no studies of the association between CST and combined cognitive and physical function.

In the ULSAM, higher GS was correlated with well-preserved cognition at baseline, which is in line with other studies on very old subjects [13, 35–37], and confirmed by a meta-analysis [33]. One explanation of this relationship can be white matter lesions or neurodegeneration in brain areas involved in both gait and cognitive functions [38]. However, we could not confirm the results from other longitudinal reports showing an association between high GS and lower risk of cognitive decline [34, 35, 39]. One reason for this may be loss to follow-up, i.e. men with incident cognitive impairment declined to participate in the MMSE at follow-up. HGS was not associated with cognition in the present study and previous results are inconsistent. Two different cross-sectional studies on old women showed opposite results [13, 40]. In the Leiden 85-plus Study, low HGS was cross-sectionally correlated with a lower MMSE and also predicted an accelerated decline in MMSE-score four years later [41]. Furthermore, a review of longitudinal studies showed an association between low HGS and decline in cognition [31]. In a US study in nonagenarians slower CST was cross-sectionally associated with dementia [37], while no association was seen between slower CST at baseline and dementia 2.6 years later [42]. Walking and chair-rising are complex activities that depend on more than just strength. Thus, GS and CST might be more sensitive than HGS to cognitive function and also to skeletomuscular disorders, such as arthritis. Further studies are needed on CST, which is a functional measure that is less investigated than GS and HGS. In the present study, higher GS was associated with independency in personal care five years later, which is in line with previous reports [32, 43–45]. Other longitudinal reports, but not our study, have also shown an association between lower GS and immobility [32, 44]. High HGS was cross-sectionally associated with independency in outdoor walking in ULSAM, which is in line with a previous review [31]. Although other reports have shown a relationship between low HGS and subsequent dependence in personal ADL the associations were not significant in our study [31, 41, 43]. In an American study, low GS and HGS were associated with institutionalisation six years later, independently of chronic conditions [43]. This is in contrast to our current findings in the ULSAM cohort, which show associations between low HGS, but not low GS, and institutionalisation five years later, also independently of comorbidity. One explanation of the different findings may be the high mean GS in the present population. CST was not associated with any of the single components of independent ageing. No other studies of the association between CST and institutionalization or disability in ADL and mobility in the oldest old have been found.

Regarding muscle mass, similar to our study the cross-sectional study by Tolea et al. found no association between low muscle mass measured by bio-electrical impedance analysis (BIA) and the combination of physical and cognitive impairment [15]. The MEDIS study reported that higher SMI was cross-sectionally associated with successful ageing [17]. However, that study included both genders, with an age range of 65–100 years. Furthermore, SMI was equation-based and the successful ageing index added together 10 components including education, BMI, physical activity and social participation, among others. With this heterogeneity in outcomes it is difficult to compare the results. No longitudinal study with a similar outcome was found. Previous studies on subjects with mean age ≥ 75 years have not shown any association between SMI and dementia or severe cognitive impairment [13, 36, 46]. Furthermore, SMI was not correlated with mobility in older women [47]. However, low muscle mass at mean age 77 was associated with ADL disability and institutionalisation five to seven years later for Australian community-dwelling men [48].

Muscle strength is lost more rapidly with ageing than muscle mass [49] and this can be one reason for finding an association between muscle function, but not muscle mass, and independent ageing in ULSAM. Furthermore, not just muscle size but also changes in muscle quality, i.e. architecture and biochemistry, may require attention [49]. However, muscle quality, including the amount of myosteatosis, cannot be evaluated by DXA.

When we related the composite sarcopenia diagnoses to our outcomes we noticed that severe sarcopenia, but not confirmed or probable sarcopenia, was cross-sectionally associated with loss of independent ageing and needing assistance when outdoor walking. However, severe sarcopenia was only present in 2% of the participants. To the knowledge of the authors, only two studies using the EWGSOP2 definition have, so far, been published [9, 10]. These studies investigated the prevalence of sarcopenia and its association with mortality and risk factors in populations with a mean age around 75 years. Sarcopenia, as defined by the EWGSOP1 definition, was not associated with independent ageing in the present study. The only similar study found was the cross-sectional study by Tolea who showed an association between sarcopenia and combined impairment in cognitive and physical performance [15]. In one cross-sectional study sarcopenia was associated with cognitive impairment [14], while no association was found in another study [13]. The participants in both studies had a mean age > 80 years. However, one meta-analysis also including younger subjects found a correlation between sarcopenia and cognitive impairment [12]. Another meta-analysis also found an association between sarcopenia and subsequent functional decline [11]. Finally, sarcopenia at mean age 77 years was associated with ADL disability and institutionalisation five to seven years later for community-dwelling Australian men [48]. However, comparisons may be slightly hampered due to somewhat different definitions used.

The prevalence of sarcopenia differs by definition criteria but also by age and gender [50]. When using the EWGSOP1 definition the prevalence in ULSAM, as well as for men in the Newcastle 85+ Study, was actually the same, 21% [51]. . In Italian octogenarian men the prevalence was 17% [52], while 13% of Belgian octogenarian men had sarcopenia [53]. These three studies also used the EWGSOP1 definition [5], but measured muscle mass with BIA, and not DXA. Using the updated definition on confirmed sarcopenia decreased the prevalence from 21 to 20% in the present study. In the only two published studies, so far, using the up-dated definition, the prevalence was below 10%, which most likely is explained by the lower mean age in these populations [9, 10].

Regarding strengths and limitations, this cohort consisted of men of similar age and ethnic background, which limits its generalizability. On the other hand this design makes it possible to exclude age, gender and ethnicity as confounders. The study population was skewed toward a healthier population than the background population, i.e. only 5% had dementia at baseline, which is lower than expected in this age group. This further reduces the generalizability. However, this might lead to underestimation of the associations observed, rather than overestimation. Misclassification is a possibility as some of the components for independent ageing were self-reported, but this potential introduction of bias was reduced by reviewing the medical records for consistency with the self-reported information. Participants who lacked information on MMSE at follow-up might also have been wrongly classified as independently aged. Although the associations were adjusted, other possible confounders, such as depression [54], might not have been taken into consideration. Finally, it is not possible to draw any conclusions about the direction of the associations between exposure and outcome in the cross-sectional analysis. However, in the longitudinal analysis of participants with independent ageing at baseline, a high GS or HGS predicted independent ageing five years later.

GS was measured with a dynamic start giving a high mean GS compared to other studies in the same age group [55]. Also, the effect of HGS might be affected as both hands, and not only the dominant, were measured and the highest value was used. This might lead to a false and excessively low prevalence of sarcopenia but also an underestimation of the associations with sarcopenia, GS and HGS.

In conclusion, muscle function, but not muscle mass, was associated with independent ageing in very old men. When using the updated EWGSOP2 definition, severe, but not probable or confirmed sarcopenia, was associated with loss of independent ageing. Otherwise, none of the present definitions of sarcopenia according to EWGSOP were associated with independent ageing. Measuring GS, HGS and CST is easy and possible to do in the clinical setting. Those assessments may identify individuals who can benefit from interventions that might at least postpone the loss of independence. However, further studies are needed to identify these possible interventions.

## Abbreviations

ADL: Activities of daily living
BIA: Bio-electrical impedance analysis
BMI: Body mass index
CI: Confidence interval
CST: Chair stand test
DXA: Dual energy X-ray absorptiometry
EWGSOP: European Working Group on Sarcopenia in Older People
GS: Gait speed
HGS: Hand grip strength
MMSE: Mini-Mental State Examination
OR: Odds ratio
SD: Standard deviations
SMI: Skeletal muscle index
ULSAM: Uppsala Longitudinal Study of Adult Men
